# How social media use and abuse are related to social cognition and loneliness in older adults

**DOI:** 10.1007/s10433-026-00925-w

**Published:** 2026-05-13

**Authors:** Qiuyi Kong, Ted Ruffman, Louise H. Phillips, Min Hooi Yong

**Affiliations:** 1 Bay Area School of Applied Psychological Sciences, Beijing Normal University at Zhuhai, Zhuhai, China; 2 Beijing Key Laboratory of Applied Experimental Psychology, National Demonstration Center for Experimental Psychology Education (Beijing Normal University), Faculty of Psychology, Beijing Normal University, Beijing, China; 3 Department of Psychology, University of Otago, Dunedin, New Zealand; 4 School of Psychology, University of Aberdeen, Aberdeen, UK; 5 Department of Psychology, School of Law and Social Sciences, University of Bradford, Bradford, UK

**Keywords:** social media use, older adults, loneliness, social cognition, theory of mind

## Abstract

Social media (SM) offers older adults new avenues for social interaction, yet its relations to social cognition remain unclear given its fundamental differences from face-to-face communication. This study examined how SM use and abuse relate to social cognition, cognitive functioning, and loneliness in older adults. A total of 529 participants aged 60–89 completed online measures of social cognition, cognitive functioning, loneliness, and social support, along with assessments of SM use and abuse. Four main findings emerged. First, greater SM use and SM abuse were both associated with poorer social cognition. Second, SM abuse, but not general use, was associated with lower cognitive functioning, particularly among adults aged 65 and older. Third, SM use was related to reduced loneliness via higher social support; however, loneliness also strengthened the association between SM use and SM abuse. Fourth, women tended to both use and abuse SM to a greater extent than men. These results suggest that SM might be a double-edged sword for older adults: moderate use is related to social connectedness and lower loneliness, whereas excessive or addictive use is linked to declines in cognitive and social functioning. The findings highlight the need to distinguish between normal and problematic SM engagement and to promote healthy digital habits among aging populations.

**Supplementary Information:**

The online version contains supplementary material available at 10.1007/s10433-026-00925-w.

## Introduction

Aging is accompanied by both positive and negative changes. Positive changes include increased resilience (Tricä et al. 2024), and relationship satisfaction, which peaks at age 65 (Buehler et al. 2021), and the well-known positivity effect (Carstensen & DeLiema 2018). However, aging also involves sub-optimal changes such as smaller social networks (Lang 2000), increased loneliness (Hawkley et al. 2019; Steijvers et al. 2024), brain decline (Casey et al. 2021; Yoo 2022), and cognitive decline (Kuiper et al. 2016). This decline in social wellbeing is accompanied by changes in cognitive functioning, including worse fluid intelligence, processing speed and working memory (Salthouse 2018). At the same time, there are declines in social cognition, including emotion recognition (e.g., Hayes et al. 2020; Ruffman et al. 2008) and theory of mind (Henry et al. 2013).

The co-occurrence of these findings suggests that a number of variables might be linked. For instance, social network decline could lead to declines in social cognition, cognitive functioning, and social wellbeing. However, it is possible that older adults’ increasing involvement in social media (SM) might offset social network decline and subsequent sequelae. SM usage among older adults aged 65 or over in the UK has increased in recent years (Department for Digital, Culture, Media & Sport, 2020). Studies suggest that approximately 2.4 million adults in this age group in the UK now use the internet, with around two-thirds of them using SM at least monthly (Age UK, 2025). This highlights the growing relevance of SM in later life, making it important to examine whether older adults’ SM usage is associated with positive or negative outcomes. We therefore examined whether SM was associated with social cognition, cognitive functioning, and loneliness in older adults.

### SM and social cognition

SM platforms are designed to support relationship maintenance, social exchange, and ongoing access to others’ views, emotions, and behaviours (Boyd & Ellison 2007; Ellison & Boyd, 2013). These platforms have transformed everyday social communication and expanded the volume and immediacy of social information available to users (Firth et al. 2019).

continues from the above sentence As a result, SM use may be closely tied to social cognition, an ability to perceive, interpret, and respond to socially relevant information (e.g., Meshi and Ellithorpe 2021; Stieger et al. 2024). On the one hand, more SM use might be associated with better social cognition. On the other hand, more SM use might be associated with worse social cognition. This could be the case either because those who go onto social media have struggled with real-life social interactions because of poorer social cognition (Jouhki et al. 2022), or because social media can impair social cognition if it replaces face-to-face contact, and given the restricted feedback available for each social exchange on social media. With regard to the latter, even video chatting over social media is not deemed a fully social process (Doheny & Lighthall 2023). However, previous research has yielded mixed findings regarding the relation between SM and social cognition, and more importantly, all such studies have been carried out with youth or young adults rather than older adults. Given that relevant research in older adults is still lacking, we first review findings from younger populations, as well as related evidence on internet use more broadly, to provide a basis for interpreting the present study.

Studies in younger adults have produced mixed findings, particularly when social cognition is assessed through self-ratings. For example, Gentina et al. (2021) found that internet use was correlated with more *positive* self-ratings in 13- to 18-year-olds, arguing that internet use might teach youth about the persuasive intent of advertising, and so, improve theory of mind. Stieger et al. (2024, Study 2) found that passive SM use (such as browsing others’ content without interaction), but not active SM use (e.g., posting, commenting, or messaging), correlated with better self-ratings of emotional intelligence. Novak et al. (2023) found a weak correlation between SM disorder in adults and a self-rated measure of alexithymia (*r* = 0.15), such that SM disorder related to more self-reported difficulty identifying own feelings. In sum, these three studies suggest a possible relation between SM and social cognition, but are contradictory. Moreover, self-ratings may not accurately reflect true social cognition.

Studies using objective measures have also yielded mixed results on the association between SM and social cognition. These studies have found either that SM is unrelated to social cognition, or is related to worse social cognition. Stewart and Kirkham (2022) used the Eyes task to measure social cognition in which participants are asked to identify the emotion or cognition present in photographs of the eyes. They found no relation with general SM use. Novak et al. (2023) and Stieger et al. (2024) also found no correlation between general SM use and social cognition in young adults. However, some studies, such as Engelberg and Sjöberg (2004), found that general internet addiction was related to worse recognition of emotional expressions in faces and verbal passages. In another study, Scarpulla et al. (2023) found that both active and passive SM use correlated with worse emotion recognition in the face, voice and body, in a group of adults. Also, Stieger et al. (2024, Study 3) found that passive SM use and SM abuse (but not active SM use) correlated with worse emotion recognition. Similarly, Ünal-Aydin et al. (2020) found that SM abuse was linked to worse Eyes task performance in young adults.

Overall, these studies suggested an inconsistent relation between SM and social cognition, but of the studies that indicated a relation, three found that SM abuse was related to worse social cognition and two that passive SM use was related to worse social cognition, and none that active SM use was related to worse social cognition. One possible reason for these inconsistent findings is that prior research has focused mainly on the association between SM use and social cognition, without considering related factors such as cognitive functioning and loneliness. Further, we note that all such studies have been carried out with youth or young adults rather than older adults. For this reason, it is uncertain whether there is a relation between SM and social cognition in older adults, so that this formed a major aim of our study.

### SM and cognitive functioning

Researchers have also examined the relation between SM use and general cognitive functioning (e.g., Firth et al. 2019; Kim & Kim 2014), which refers to abilities involved in information processing such as attention, working memory, and executive control. Some research is suggestive of a possible positive effect for SM on cognition in older adults, but it is sometimes contradictory or doesn’t show a clear effect.

In particular, some observational studies have suggested a possible positive association between SM use and cognitive functioning in older adults. For example, Khoo and Yang (2020) measured frequency of SM use and a range of executive function abilities in over 3500 middle-aged and older adults, and found that SM was linked to social support through family, which in turn was linked to perceived constraints and then executive function. In another study, Kim and Kim (2014) found that SM users had a slightly higher score on the Mini Mental State Exam, a measure used to screen for dementia, although we note that this is a rather coarse measure of cognition.

Intervention studies have also provided limited and somewhat mixed support for a positive effect of SM on cognitive functioning in older adults. Quinn (2018) examined SM by randomly placing participants in either a control or intervention group, with the latter group receiving eight hours of teaching regarding how to use SM. They found that the intervention group experienced a greater gain in inhibitory ability (reduced time on a Stroop task), with no differences on the other measures. In another intervention study, Mhyre et al. (2017) randomly assigned participants to a Facebook® or control group, and found that the Facebook group had a significantly greater gain on one cognitive task after a seven-week period, although not on the other 11 tasks. This rather weak result, coupled with the very low *N* (only 14 participants in each group), limits the impact of this study.

Overall, while some studies suggest a possible positive effect of SM, findings remain inconsistent. This led the authors of a recent review of 21 studies to conclude that individual studies were often of poor quality and that “(t)he relationship between (SM) use, wellbeing and cognitive function was inconclusive” (Newman et al. 2021, p.377).

### SM and loneliness

Given the inherently social nature of SM, older adults’ SM use might influence social connection, perceived support, and feelings of loneliness. However, existing findings are mixed, and it remains unclear whether SM use is associated with higher or lower loneliness in later life. Rolandi et al. (2020) compared a group of older adults who had been trained to use SM to an untrained group, and found that those in the trained group used SM more, had a smaller reduction in social contacts, and had a reduced feeling of being left out during the COVID-19 lockdown in 2020. Zhang et al. (2021) found that older adults’ SM communication was linked to perceived social support and social contact, which in turn, mediated a link to lower loneliness. Quinn (2021) further showed that older participants assigned to an SM training group experienced a larger decrease in loneliness than controls. However, findings are not entirely consistent. Hutto et al. (2015) found that passive SM use was not associated with loneliness, whereas active SM use was associated with reduced loneliness. In addition, Aarts et al. (2015) found no indication that SM use in older adults was related to loneliness.

Given these inconsistent findings, several reviews have attempted to clarify the relation between SM use and loneliness in older adults. Kusumoto et al. (2022) reviewed 11 studies and concluded that 64% of studies linked SM to reduced loneliness. Wiwatkunupakarn et al. (2022) examined 15 studies and found very limited evidence for the link between SM and loneliness/depression in experimental studies. Similarly, Cotten et al. (2022) concluded that the relationship between SM and measures of wellbeing such as loneliness is not well understood, and Lei et al. (2024) examined 64 studies and determined that few conclusions could be reached given the small number of longitudinal and intervention studies, and the variations in participants, measurement of SM use, and outcome measures.

In summary, the research described above is contradictory and inconclusive as to whether SM is associated with increased or reduced loneliness in older adults, possibly because of differences in how SM use is measured and variations in research methods. To address this gap, the present study examined loneliness, as well as real-life social support, alongside social cognition and cognitive functioning in older adults.

### Present study

We had several aims in the present study. These included examining whether SM use in older adults is related to (a) better or worse social cognition, (b) better or worse cognitive functioning, (c) better or worse social wellbeing as indexed by measures of loneliness and social support. To further clarify these associations, we also examined whether the relation between SM use and social cognition remained after accounting for key demographic variables and cognitive functioning, and whether the relation between SM use and loneliness was mediated by social support.

In addition, whereas prior studies have primarily focussed on SM use, we also considered SM abuse. Abuse consists of addictive behavior on SM, such as habitual checking of SM, or anxiety over withdrawal from SM (van den Eijnden et al. 2016). This distinction is important because excessive SM engagement may relate to different outcomes than SM use itself. As reviewed above, problematic or disordered SM use is associated with poorer social-cognitive performance (Ünal-Aydin et al. 2020) and with difficulties in emotional awareness (Novak et al. 2023) in young adults. Previous research also indicates that there are general links between addictive behavior and lower executive functions (Salmani et al. 2020). It seems plausible, then, that older adults with SM abuse (a type of addiction) might have greater difficulties in cognitive functioning, which might then contribute to worse social cognition and higher loneliness.

Given age-related changes in social cognition and cognitive functioning across later life, we also considered age as an important factor. Participants were therefore divided into two age groups using 65 years as the cutoff, allowing us to examine whether these associations differed across stages of older adulthood. We selected 65 years old as the cut-off point because (1) significant declines in cognitive functioning occur after the age of 65 (e.g., Petersen et al. 2018); and (2) the age of 65 is widely viewed as the start of old age in real life (e.g., linked to the default retirement age and eligibility for benefits). In addition, given the potential role of gender on SM use (e.g., Mari et al. 2023), we conducted analyses of gender effects.

## Method

### Participants

Participants were 529 older adults (244 men, 281 women, 1 non-binary, and 3 with missing gender data) aged 60–89 years (*M* = 65.87, *SD* = 5.15). The majority (93.4%) reported English as their first language; the remainder were non-native but proficient English speakers. All participants were recruited from the United Kingdom via the Prolific platform (https://www.prolific.com/). Ethnic composition was 96.4% European, 1.5% Asian, 1.1% African, and 1.0% other. Education was rated on a 7-point Likert scale (0 = no formal qualifications, 1 = secondary education, 2 = high school diploma, 3 = technical or community college, 4 = undergraduate degree, 5 = graduate degree, 6 = doctorate), with a mean of 3.23. None of participants reported diagnoses of vision, hearing, or cognitive impairment (e.g., Alzheimer’s disease, autism, or language-related disorders). The data were collected over two separate sessions and not all participants completed both days for all tasks. As such, valid data were available for 458 participants on SM abuse and for 479 participants on SM use. To further examine age-related differences, we divided participants into two age groups: young-old (60–64 years) and old-old (65 + years). Each age group had a near-even number of participants (young-olds: *n* = 259, old-olds: *n* = 270).

We used G*Power (Faul et al. 2007) to calculate the required sample size for the correlations and regressions in our study. For the correlation analysis (two-tailed, effect size = 0.30, *α* = 0.05, and power = 0.95), the results indicated a required sample size of 138. For the linear multiple regression analysis (effect size = 0.15, *α* = 0.05, power = 0.95, number of tested predictors = 3, and number of predictors = 6), the analysis resulted in a required sample size of 119. Because some analyses were conducted separately for the young-old and old-old groups, this minimum sample size needed to be achieved within each group. We recruited a larger sample because larger samples have the advantage of helping to identify smaller effects, reduces the margin of error, results in higher confidence levels, and models the data with more accuracy (Gumpili & Das 2022). Nevertheless, to avoid the obvious shortcoming of larger datasets (achieving significance with very small effect sizes), sensitivity analyses based on randomly selected subsamples of 150 participants from each of the young-old and old-old group were also used. These are reported in the Supplementary Materials and showed the same pattern of results. Only the full-sample results are reported in the main text. Importantly, no statistical analyses were conducted before data collection had been completed.

### Materials

#### SM use

We assessed SM use via a four-item checklist covering the past three months. Participants indicated whether they had (a) used instant-messaging apps (e.g., WhatsApp, Facebook Messenger, Telegram); (b) participated on social-networking sites (e.g., Facebook, Instagram, Twitter); (c) expressed opinions on these sites (e.g., commenting, posting, retweeting); and (d) uploaded content (e.g., photos, videos, blog posts). Each “yes” response was scored 1. Total scores range from 0 to 4, with higher scores indicating a broader range of SM activities.

#### SM abuse

We assessed problematic SM abuse with the nine-item Social Media Disorder Scale (van den Eijnden et al. 2016). Participants rated each item (e.g., “During the past year, have you tried to spend less time on social media, but failed?”) on a 5-point Likert scale from 1 = Strongly Disagree to 5 = Strongly Agree. Each of the nine items corresponds to one criterion of problematic SM use: preoccupation, tolerance, withdrawal, displacement, escape, problems, deception, displacement, and conflict. The average score was used in the analysis, with higher scores indicating greater SM abuse. In the present sample, the scale demonstrated excellent internal consistency (*α* = 0.893).

#### Social cognition

We used the Reading the Mind in the Eyes task (Baron-Cohen et al. 2001) to assess older adults’ social cognition. The task comprises 36 black-and-white photographs of the eye region, with each photo accompanied by four words positioned at the corners of the screen. Participants were required to select one out of the four words that best described the depicted individual’s feelings or thoughts. The total number of correct responses (maximum = 36) was used in the analyses.

#### Cognitive functioning

We assessed older adults’ cognitive functioning through four well-validated tasks that measure working memory, processing speed, attention, and inhibition. These tasks included (1) 2-back, (2) verbal fluency, (3) backward digit-span, and (4) colour-shape switching.

*2-Back Task* We used a 2-back task to measure older adults’ ability to monitor and update information in their working memory (Jaeggi et al. 2008). Participants were presented with a sequence of numbers and required to indicate whether the current stimulus matched the one that appeared two positions back in the sequence. For example, in the sequence 3–7–3, the third number is a target because it matches the number shown two trials earlier. We recorded the total number of correct trials, with a maximum of 100.

*Verbal Fluency Task* We used the verbal fluency task to assess older adults’ lexical access, semantic memory, divergent thinking and inhibitory control (Gajewski et al., 2018; Lezak et al. 2012). Participants were asked to generate as many words as possible within 60 s based on two rules: (1) words starting with the letter “A”, and (2) words belonging to professions. The participant's score was the total number of unique correct words.

*Backwards Digit Span Task* We used a backwards digit-span test to measure older adults’ attention and working memory (Wechsler et al., 2008). There were 24 trials in which participants were auditorily presented with a sequence of digits (2–8 items) and required to immediately repeat the sequence in reverse order. The total number of correct trials was recorded, with a maximum score of 24.

*Colour-Shape Switching Task* We used a colour-shape switching task to assess older adults’ cognitive flexibility and switching ability (Miyake et al. 2004). Participants viewed a series of coloured geometric shapes (blue or green rectangles and squares) and were required to indicate the relevant feature (colour or shape) according to specific cues. The task included three blocks: a shape-only, a colour-only, and a mixed-cue block. Block 3 was a mixed-cue block with 130 trials, equally divided between repeated-cue (colour → colour, shape → shape) and switched-cue (colour → shape, shape → colour) trials. This task taps flexibility and switching ability because participants had to shift between response rules and inhibit the previously relevant rule. The total number of correct trials was used in the analysis.

Since the four executive function tasks were significantly correlated with each other (with one correlation significant at *p* = 0.03, and five others at *p* < 0.001), we created a composite score of cognitive functioning by *z*-standardising the raw scores from each task and then averaging them. This approach is widely used in cognitive aging research to capture shared variance across tasks and provide a more reliable estimate of overall cognitive functioning (e.g., Langbaum et al. 2020; Lim et al. 2016).

#### Loneliness

We used the six-item Loneliness Scale to assess loneliness (De Jong Gierveld & van Tilburg 2006). The scale comprises three emotional-loneliness items (e.g., “I experience a general sense of emptiness”) and three social-loneliness items (e.g., “There are enough people I feel close to”, reverse-coded). Participants rated each item on a 3-point scale (1 = no, 2 = more or less, 3 = yes), with higher scores indicating greater loneliness. As there was no clear theoretical basis for analysing the two subdomains separately, loneliness was treated as a single construct. The Cronbach’s *α* for this sample was 0.805.

#### Social support

We assessed participants’ social support using the six-item short form of the Lubben Social Network Scale (Lubben 1988; Lubben et al. 2006). Participants indicated, on a 6-point scale (0 = none, 1 = one, 2 = two, 3 = three or four, 4 = five to eight, 5 = nine or more), how many relatives and friends (a) they saw or heard from at least once a month, (b) with whom they felt at ease discussing private matters, and (c) to whom they felt close enough to ask for help. The scale had excellent internal consistency (*α* = 0.817).

### Procedure

Participants were tested on the online Gorilla research platform with tasks given in this study part of a larger study on reading habits. The tasks were presented in a fixed order, with the items in each scale randomized. Participants received a small sum for their participation. The study was approved by the University Ethics Panel (reference number: E1157) and carried out in accordance with American Psychological Association ethical guidelines. Written informed consent was obtained from all participants before testing began.

### Transparency and openness

We report how we determined our sample size, all data exclusions, all manipulations, and all measures in the study, and we follow JARS (Appelbaum et al. 2018). Data were analyzed using the SPSS statistical package. All data and research materials are available at: https://osf.io/h2wyd/files/63q8y.

## Results

### Analysis plan

We first present the descriptive statistics and correlations among the main variables in all participants. We then compared the two age groups on their social cognition, cognitive functioning, loneliness, and SM use and abuse, and computed correlations among these measures separately within each age group. Next, we used regressions to examine whether SM use and SM abuse predicted changes in social cognition and cognitive functioning in the two age groups. Finally, we examined whether SM use reduced older adults’ loneliness through increased social support, and how SM use and loneliness were related to SM abuse.

### Descriptives and correlations

Table 1 lists the descriptive statistics and correlations among the main variables for all participants. The correlation analysis revealed three main findings. First, more SM use was correlated with worse social cognition, and also with a lower level of loneliness and more social support. Second, SM abuse was associated with worse cognitive functioning, lower social cognition, and higher loneliness. Last, there was a positive correlation between SM use and SM abuse.

**Table 1:** Descriptive Statistics and Between-Person Correlations in All Participants

		Descriptives		Correlations								
	*n*	*M*	*SD*	1	2	3	4	5	6	7	8	9
1. Age	523	65.87	5.16	–								
2. Gender	526	–	–	0.031	–							
3. Education	529	3.23	1.47	0.004	−0.004	–						
4. Cognitive Functioning	529	0.00	0.62	−0.081	0.016	**0.108**^**a**^	–					
5. Social Cognition	529	25.85	4.65	−0.036	−0.048	0.060	**0.238**^**c**^	–				
6. Loneliness	529	2.44	0.76	**−0.089**^**a**^	0.015	−0.007	−0.079	−0.058	–			
7. Social Support	528	13.98	5.60	0.083	**−0.122**^**b**^	0.055	0.068	0.034	**−0.513**^**c**^	–		
8. SM Use	479	2.73	1.24	−0.083	**−0.117**^**a**^	0.020	0.047	**−0.123**^**b**^	**−0.106**^**a**^	**0.148**^**b**^	–	
9. SM Abuse	458	1.88	0.82	**−0.154**^**b**^	**−0.191**^**c**^	0.010	**−0.105**^**a**^	**−0.193**^**c**^	**0.160**^**c**^	−0.030	**0.333**^**c**^	–

Means, standard deviations, and Pearson correlations are presented in the table. Gender: women = 0, men = 1. Significant correlations are shown in bold. ^a^*p* < 0.05, ^b^*p* < 0.01, ^c^*p* < 0.001.

Table 2 lists differences between young-old (60–64 years) and old-old (65 + years). Independent-samples *t*-tests revealed that compared to young-olds, old-olds had a lower level of loneliness, *t*(527) = -2.09, *p* = 0.037, *d* = 0.758, and a smaller tendency towards SM abuse, *t*(456) = -2.21, *p* = 0.027, *d* = 0.813. Otherwise, the age groups showed no significant differences in cognitive functioning, *t*(527) = -2.09, *p* = 0.614, *d* = 0.624, social cognition,* t*(527) = 0.099, *p* = 0.921, *d* = 4.65, or SM use, *t*(477) = 0.382, *p* = 0.703, *d* = 1.24.

**Table 2:** Descriptive Statistics and Correlations Among Main Variables in Young-Old (Above the Diagonal) and Old-Old (Below the Diagonal)

	Young−old (*n* = 259)		Old−old (*n* = 270)		Correlations								
	*M*	*SD*	*M*	*SD*	1	2	3	4	5	6	7	8	9
1. Age	61.84	1.47	69.81	4.36	–	0.054	0.060	−0.092	−0.073	0.001	0.013	−0.035	−0.051
2. Gender	–	–	–	–	**0.162**^**b**^	–	−0.020	0.090	−0.093	0.045	**−0.143**^**a**^	**−0.139**^**a**^	**−0.232**^**c**^
3. Education	3.27	1.46	3.19	1.49	0.034	0.010	–	0.059	−0.001	−0.021	0.004	0.006	−0.003
4. Cognitive Functioning	0.01	0.64	−0.01	0.61	**−0.125**^**a**^	−0.064	**0.156**^**a**^	–	**0.236**^**c**^	−0.080	0.037	0.014	−0.045
5. Social Cognition	25.83	4.32	25.87	4.96	−0.062	−0.009	0.109^†^	**0.241**^**c**^	–	−0.093	−0.011	**−0.143**^**a**^	**−0.208**^**b**^
6. Loneliness	2.51	0.76	2.38	0.76	−0.038	−0.027	0.002	−0.083	−0.029	–	**−0.533**^**c**^	−0.064	0.104
7. Social Support	13.50	5.54	14.43	5.64	0.028	−0.092	0.105^†^	0.103^†^	0.071	**−0.486**^**c**^	–	0.073	−0.020
8. SM Use	2.70	1.22	2.75	1.26	**−0.204**^**b**^	−0.095	0.033	0.081	−0.108^†^	**−0.144**^**a**^	**0.216**^**c**^	–	**0.406**^**c**^
9. SM Abuse	1.97	0.86	1.80	0.77	**−0.150**^**a**^	**−0.161**^**a**^	0.022	**−0.182**^**b**^	**−0.177**^**b**^	**0.203**^**b**^	−0.024	**0.266**^**c**^	–

Means, standard deviations, and Pearson correlations are presented in the table. Gender: women = 0, men = 1. In the young-old group, there were 127 women (49.0%), 131 men (50.6%), and 1 non-binary participant (0.4%). In the old-old group, there were 117 women (43.3%) and 150 men (55.6%); 3 responses for gender were missing (1.1%). Significant correlations are shown in bold. ^†^*p* < 0.10, ^a^*p* < 0.05, ^b^*p* < 0.01, ^c^*p* < 0.001.

Table 2 lists the correlations among main variables separately in young-old and old-old. For young-old, SM use and SM abuse were only correlated with gender (SM use:* r* = -0.139, *p* = 0.033, SM abuse: *r* = -0.232, *p* < 0.001) and worse social cognition (SM use:* r* = -0.143, *p* = 0.028, SM abuse: *r* = -0.208, *p* = 0.002). For old-old, however, SM use was related to worse social cognition (marginally significant; *r* = -0.108, *p* = 0.093), lower loneliness (*r* = -0.144, *p* = 0.025), and more social support (*r* = 0.216, *p* < 0.001), whereas SM abuse was correlated with gender (*r* = 0.162, *p* = 0.008), worse cognitive functioning (*r* = -0.182, *p* = 0.006), lower social cognition (*r* = -0.177, *p* = 0.007), and higher loneliness (*r* = 0.266, *p* < 0.001).

Overall, SM abuse, but not SM use, had a negative relation with older adults’ cognitive functioning, social cognition and loneliness, with those over 65 years old particularly vulnerable. Also of note, women in both groups were more likely to abuse SM than men, and young-old women were more likely to use SM compared to young-old men. These gender patterns remained unchanged after controlling for age, particularly for SM abuse in both groups.

### SM and cognitive functioning

Next, we examined whether SM use and abuse were significantly associated with cognitive functioning in young-old and old-old adults. Given the correlation between SM abuse and cognitive functioning in the old-old group, we ran two separate hierarchical regressions with cognitive functioning as the outcome variable. The first regression was conducted for the young-old group, and the second for the old-old group. In both analyses, we entered age, gender and education as covariates in Step 1, and entered SM use and abuse simultaneously in Step 2. We examined tolerance and variance inflation factor (VIF) values for all predictors to assess multicollinearity. These values were well within commonly accepted thresholds (tolerance > 0.20, VIF < 5), indicating no evidence of multicollinearity.

Table 3 presents the results of regressions predicting cognitive functioning in the two age groups. For the young-old, cognitive functioning was not predicted by either SM use or SM abuse. For the old-old, however, cognitive functioning was significantly predicted by SM use and abuse together, *∆R*^*2*^ = 0.056, *F*_*change*_(2, 219) = 6.69, *p* < 0.01. When examining the unique effects of SM use and abuse in the old-old group, SM abuse was a unique predictor of cognitive functioning, *β* = -0.237, *t* = -3.47, *p* < 0.001, whereas SM use was marginally significant, *β* = 0.129, *t* = 1.92, *p* = 0.056.

**Table 3:** Regression Predicting Cognitive Functioning in Young-Old and Old-Old Adults

	Young-old			Old-old		
	*β*	*p*	*∆R*^*2*^	*β*	*p*	*∆R*^*2*^
*Step 1*			0.016			0.027
Age	−0.103	0.121		−0.085	0.204	
Gender	0.070	0.292		−0.019	0.778	
Education	0.031	0.637		0.142	0.034	
*Step 2*			0.003			0.056^b^
Age	−0.103	0.123		−0.097	0.146	
Gender	0.063	0.355		−0.056	0.396	
Education	0.029	0.660		0.145	0.026	
SM Use	0.046	0.532		0.129	0.056	
SM Abuse	−0.053	0.472		−0.237	< 0.001	

Age, gender, and education were entered in Step 1 as control variables. *β* represents the standardized regression coefficient, and *∆R*^*2*^ refers to the additional variance explained by the variables added at each step. ^b^*p* < 0.01.

### SM and social cognition

We then used two separate regressions (Table 4) to examine whether SM use and abuse were significantly associated with social cognition in the two age groups. In each regression, we entered control variables (age, gender, and education) in Step 1, and then two predictors (SM use and abuse) in Step 2. Building on prior literature indicating the relationship between social cognition and cognitive functioning in older adults (e.g., Horning et al. 2012; Murphy et al. 2019) and our correlational findings (see Table 2), we also entered cognitive functioning in Step 3 to examine whether the relation between SM use/abuse and social cognition remained after accounting for demographic variables and cognitive functioning. In the young-old group, SM abuse showed a significant negative association with the outcome, *β* = -0.217, *t* = -3.01, *p* < 0.01, which remained significant after accounting for cognitive functioning, *β* = -0.203, *t* = -2.92, *p* < 0.01, whereas SM use was not significantly associated. In contrast, in the old-old group, SM use remained significantly associated with the outcome after controlling for cognitive functioning, *β* = -0.208, *t* = -3.15, *p* < 0.01, while the association with SM abuse was no longer significant. Cognitive functioning was a strong positive correlate in both groups.

**Table 4:** Regression Predicting Social Cognition in Young-Old and Old-Old Adults

	Young−old			Old−old		
	*β*	*p*	*∆R*^*2*^	*β*	*p*	*∆R*^*2*^
*Step 1*			0.017			0.008
Age	−0.084	0.207		−0.032	0.641	
Gender	−0.093	0.160		0.060	0.378	
Education	0.026	0.697		0.059	0.383	
*Step 2*			0.063^c^			0.066^c^
Age	−0.097	0.134		−0.078	0.242	
Gender	−0.153	0.022		0.028	0.675	
Education	0.027	0.679		0.061	0.350	
SM Use	−0.077	0.276		−0.176	0.010	
SM Abuse	−0.217	0.003		−0.157	0.024	
*Step 3*			0.063^c^			0.057^c^
Age	−0.071	0.261		−0.054	0.408	
Gender	−0.169	0.009		0.042	0.517	
Education	0.019	0.758		0.025	0.701	
SM Use	−0.089	0.196		−0.208	0.002	
SM Abuse	−0.203	0.004		−0.097	0.157	
Cognitive Functioning	0.254	< 0.001		0.250	< 0.001	

These results suggest that SM use and abuse were related to worse social cognition, especially in old-old adults. Further, SM use and abuse show distinct patterns across age groups, such that their associations with social cognition differ after accounting for cognitive functioning. In the old-old group, the association between SM abuse and social cognition was no longer significant after controlling for cognitive functioning, suggesting that this association may be partly accounted for by a decrease in cognitive functioning.

Age, gender, and education were entered in Step 1 as control variables. *β* represents the standardized regression coefficient, and *∆R*^*2*^ refers to the additional variance explained by the variables added at each step. ^c^*p* < 0.001.

### SM use and loneliness

Loneliness and social support were significant correlates of SM use in the old-old group and in the full sample. Therefore, our next aim was to examine whether SM use predicted loneliness through increased social support among old-old adults and across all participants. We did not conduct mediation analysis for the young-old group because loneliness, social media use, and social support were not significantly correlated in this group. Also, cognitive functioning and social cognition were not included in these analyses, as neither showed significant associations with loneliness (see Tables 1 and 2). We used a mediation analysis with the bootstrapping procedure on PROCESS macro (Hayes, 2018; Preacher & Hayes, 2008). Given that age, gender, and education level were correlates of SM use in our results (see Tables 1 and 2) and previous studies (Janssen et al. 2025), we included these variables as covariates. We generated 5,000 bootstrap samples and computed bias-corrected and accelerated 95% confidence intervals (CI) to assess the indirect effects.

**Fig. 1:**
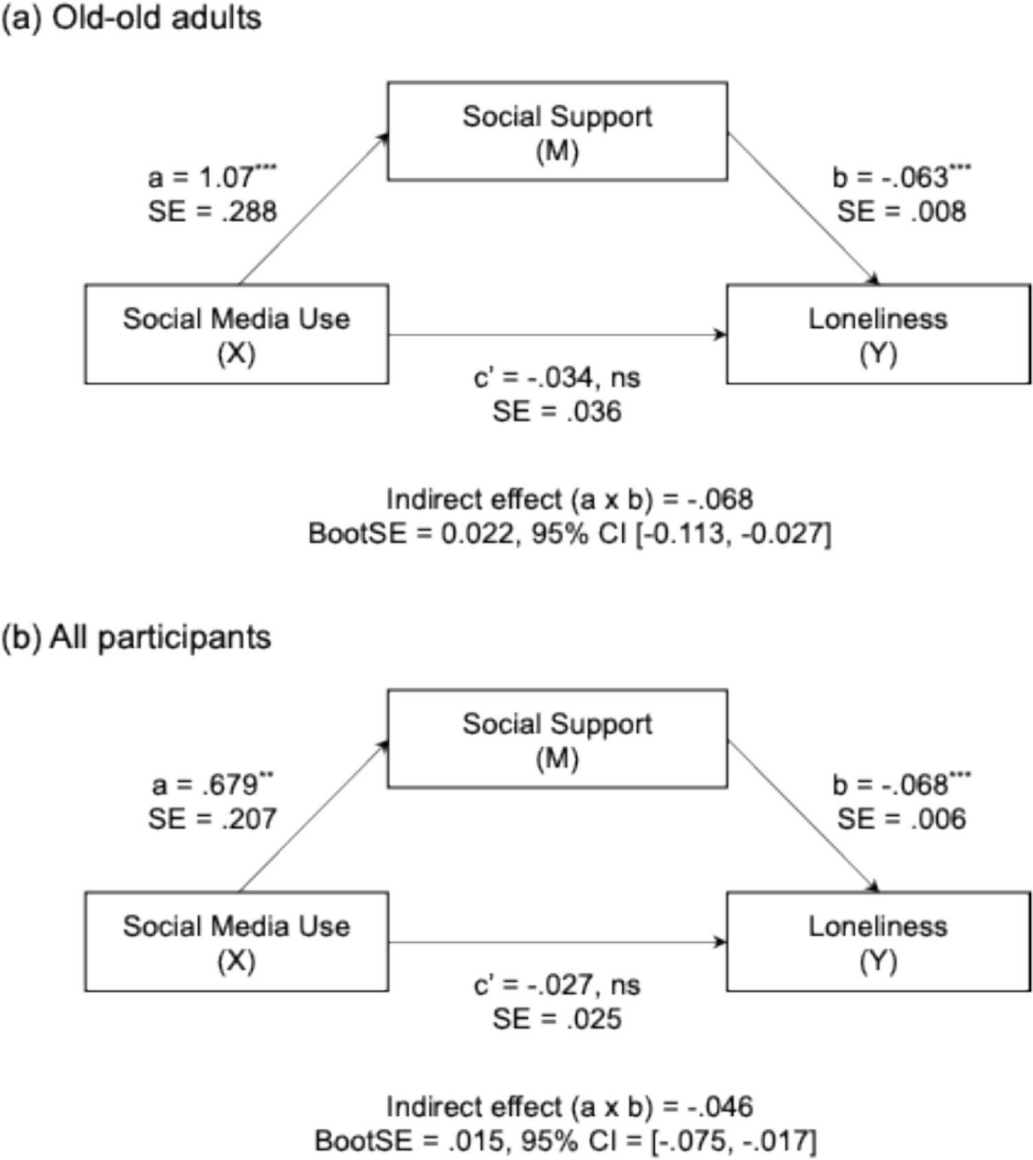
Mediating Role of Social Support in the Association between Social Media Use and Loneliness Among Old-Old Adults (a) and in the Full Sample (b). Mediation analyses were carried out after controlling for age, gender, and education. Panel (a) presents the mediation model for the old-old group, and panel (b) presents the mediation model for the full sample. Mediation analysis was not conducted for the young-old group because loneliness, SM use, and social support were not significantly correlated in that group. ns = not significant, ^**^p < 0.01, ^***^p < 0.001

In the old-old group (see Fig. 1a), the indirect effect of SM use on loneliness through social support was significant (effect = -0.068, *BootSE* = 0.022, 95% CI [-0.113, -0.027]), suggesting that greater SM use was associated with higher social support, which in turn was associated with lower loneliness in old-old adults.

In the full sample, the indirect effect of SM use on loneliness was significant (effect = -0.046, *BootSE* = 0.015, 95% CI = [-0.075, -0.017]), while the direct effect of SM use on loneliness was not significant (*β* = -0.027, *SE* = 0.025, *t* = -1.09, *p* = 0.278, 95% CI = [-0.076, 0.022], see Fig. 1b). The results showed that social supported accounted for 63.25% of the correlation between SM use and loneliness, suggesting a strong mediating role of social support on the correlation between SM use and loneliness across all participants.

Given the significant correlations among SM use, loneliness, and SM abuse among the old-old group and in the full sample, we also examined whether SM use and loneliness predicted unique variance in SM abuse among old-old adults and in all participants (see Table 5). Our goal was to explore the factors that might contribute to older adults’ SM abuse. Age, gender, and education were entered as control variables, and SM use and loneliness were centered and entered into a regression along with the SM use × loneliness interaction.

**Table 5:** Regression Predicting Older Adults’ Social Media Abuse Among Old-Old Adults and in All Participants

	*B*	*SE*	*β*	*t*	*p*	*ΔR*^*2*^
*Old-old adults*						
Age	−0.015	0.011	−0.088	−1.38	0.170	0.022
Gender	−0.267	0.094	−0.178	−2.83	0.005	0.029
Education	0.001	0.030	0.002	0.037	0.971	0.000
SM Use	0.217	0.049	0.282	4.41	< 0.001	0.054
Loneliness	0.174	0.047	0.235	3.68	< 0.001	0.046
SM Use × Loneliness	0.085	0.037	0.109	1.71	0.088	0.011
All Variables: Total *R*^2^						0.163
*All participants*						
Age	−0.019	0.007	−0.116	−2.74	0.006	0.024
Gender	−0.307	0.068	−0.191	−4.54	< 0.001	0.041
Education	−0.011	0.023	−0.020	−0.465	0.642	0.000
SM Use	0.296	0.036	0.349	8.18	< 0.001	0.104
Loneliness	0.158	0.035	0.197	4.56	< 0.001	0.033
SM Use × Loneliness	0.078	0.037	0.090	2.12	0.035	0.008
All Variables: Total *R*^2^						0.209

**Fig. 2:**
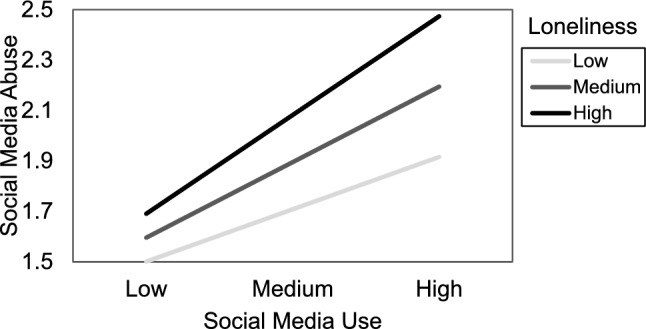
Loneliness Moderates the Relation Between Social Media Use and Social Media Abuse in All Participants. Participants rated the items of SM abuse on a 5-point Likert scale, ranging from 1 = Strongly Disagree to 5 = Strongly Agree

Among the old-old adults, SM use and loneliness showed independent associations with SM abuse, whereas the SM use × loneliness interaction was marginally significant. In the full sample, SM use, loneliness, and the SM use × loneliness interaction were unique predictors of SM abuse. The significant interaction effect in the full sample indicated that older adults’ loneliness moderated the relation between SM use and SM abuse (Fig. 2), such that the positive association between SM use and SM abuse was stronger among older adults with higher levels of loneliness.

*B, SE, β, t* and* p* values are reported with all predictors simultaneously entered into the regression equation. *ΔR*^*2*^ refers to the proportion of independent variance accounted for by each variable in the model.

## Discussion

We examined how SM use and abuse were related to social cognition, cognitive functioning, and loneliness in 529 older adults, considering whether SM use might be associated with positive effects in older adults in the absence of extensive social networks such as better social cognition, cognitive functioning and loneliness. To the best of our knowledge, the present study is the first to investigate the association between social media use and social cognition among older adults.

Our study yielded four key findings: (1) individuals who used and also abused SM tended to show poorer social cognition; (2) the negative relation between SM and cognitive functioning was confined to problematic engagement such as SM abuse, and was observed only in individuals aged 65 and older; (3) although SM use was associated with lower loneliness in older adults for those with higher social support, it was also associated with a risk of SM abuse, especially among those feeling lonely; (4) older women were more likely to use and abuse SM compared to older men. We discuss each of these findings below.

### SM use and worse social cognition

The primary aim of the present study was to examine whether greater SM use was related to better or worse social cognition in older adults. Our findings revealed that those who used SM more broadly, and particularly those who engaged in excessive use (SM abuse), tended to exhibit poorer social cognition. This pattern was consistent with that established in previous research with young adults (a negative relation). Recent social-cognitive neuroscience work suggests that remote interpersonal communication, including social media, engages core processes such as social perception, social reward, and theory of mind, supporting the expectation that SM use may be related to social cognition (Doheny & Lighthall 2023). These results suggest two possible interpretations: (1) SM use might impair social cognition or (2) poorer social cognition might predispose older adults to get their social fix through heavier SM use. Prior research on escapism and social compensation provides some support for the second interpretation, suggesting that those with poorer social cognition might turn to online social platforms as a means of compensating for limited offline social engagement (Janssen et al. 2025; Kraut et al. 1998) or avoiding their real-world shortcomings (Jouhki et al. 2022). Though our study does not definitively support one of these interpretations, it is important in being the first to show a link between SM abuse and poorer social cognition in older adults.

We also observed age-group differences in the associations between SM and social cognition. In the old-old group, the association between SM abuse and social cognition was no longer significant after controlling for cognitive functioning, but this pattern did not emerge in the young-old group. This suggests that, in old-old adults, the association between SM abuse and social cognition may be partly accounted for by cognitive functioning, a result that is consistent with prior research suggesting a link between social cognition and cognitive functioning in later life (Horning et al., 2012; Murphy et al. 2019).

### SM use and worse cognitive functioning

The second aim of the present study was to examine the relation between greater SM use and cognitive functioning in older adults, finding that SM use was not related to cognitive functioning, but SM *abuse* was linked to poorer cognitive functioning, particularly among adults aged 65 and above. These findings contrast with previous research suggesting a positive relation between SM use and cognition in older adults (e.g., Kim & Kim 2014; Mhyre et al. 2017; Quinn 2018), and also with some studies that have reported no significant relation (Khoo & Yang 2020). Our finding is interesting as it differentiates normal SM use and problematic use in older adults. However, it remains unclear whether (a) lower cognitive ability increases SM abuse, (b) SM abuse contributes to cognitive decline, or (c) a third variable underlies this association. Although the most obvious interpretation is the first – that worse cognitive functions lead to SM abuse – future research employing longitudinal or intervention designs that track SM use, social cognition, and cognitive functioning over time would be valuable for clarifying causal relationships.

### SM use and loneliness

The third aim of the present study was to examine how SM relates to older adults’ loneliness. There were both positive and negative relations. Our mediation analysis showed that SM use was associated with greater social support, which in turn reduced older adults’ loneliness. This finding aligns with previous evidence highlighting the mediating role of social support in the relation between SM use and loneliness (Zhang et al. 2021), and is consistent with other studies showing that SM use is linked to lower loneliness among older adults (e.g., Lei et al., 2025; Rolandi et al. 2020). However, we also found that SM use was associated with an increased risk of SM abuse, particularly among lonely individuals. As noted earlier, SM abuse was related to poorer social cognition and cognitive functioning in older adults. In sum, while SM use may promote social connectedness and reduce loneliness in older adults, it also carries the risk of unhealthy overuse, especially for those who are more lonely.

### SM use and gender

A final finding was that older women were more prone to SM use and abuse compared to older men. This is consistent with findings for adolescents (Twenge & Martin 2020) and young adults (Mari et al. 2023), indicating similar behavior over the lifespan. A further possibility is that these gender differences partly reflect reporting tendencies. Recent evidence indicates that social desirability can shape self-reports in complex ways (Zuo & Mao 2025). Therefore, the possibility of gender differences in reporting bias cannot be ruled out. We also note that whereas females’ SM use is associated with negative outcomes for wellbeing as indicated by loneliness amongst adolescent girls (Twenge & Martin 2020), we did not find such a clear link in our sample of older females.

### Limitations and future directions

While the present study provides the first evidence for the relation between SM use and social cognition in older adults, there are several limitations. First, we did not differentiate between active and passive SM use, although they may have differential relations with older adults’ social cognition (e.g., Stieger et al. 2024, Study 3). However, this dichotomy has been criticized for oversimplifying the complexity of online behavior (Kaye 2021). There has been considerable inconsistency in how these activities are best categorized (Kaye 2021), and there is a very plausible confound in that active SM users have better wellbeing to begin with (i.e., have the confidence to post material) whereas those who are passive users have worse wellbeing. Future research could adopt more nuanced categorizations of SM use to better capture the diverse nature of online engagement and its impacts. Second, as discussed earlier, the data were collected at a single time point, which prevents any inference about causal relations between SM use and changes in social cognition. Although our cross-sectional data offer a valuable starting point given the absence of prior research on this topic, future studies should adopt longitudinal or intervention designs to further examine these questions. Further, we did not account for broader life circumstances, such as marital status and employment status, which may also shape older adults’ social experiences. Future research should consider both online and offline social activity to provide a more comprehensive understanding of age-related changes in older adults.

### Conclusion

The present study examined how social media use and abuse were related to social cognition, cognitive functioning and wellbeing among older adults, and to our knowledge, it is the first to examine their association with social cognition in this population. The findings suggest that SM has a mixed profile; although excessive or problematic use is associated with detrimental relations with social cognition, cognitive functioning, and wellbeing, moderate engagement is associated with more positive relations with other variables. Future research should employ longitudinal or intervention designs to further clarify the causal mechanisms underlying these associations.

## Supplementary Information

Below is the link to the electronic supplementary material.

Supplementary file1 (DOCX 90 KB)

## Data Availability

Data are available from the Open Science website: https://osf.io/h2wyd/files/63q8y
