# Supplementary material for: How social media use and abuse are related to social cognition and loneliness in older adults
Source: Eur J Ageing. 2026 May 13;23(1):30. doi: 10.1007/s10433-026-00925-w (PMC13338074; doi:10.1007/s10433-026-00925-w)
Supplement: Supplementary file 1 — Supplementary file1 (DOCX 90 KB) [file 10433_2026_925_MOESM1_ESM.docx]

**Supplementary Materials for**

**How Social Media Use and Abuse are Related to Social Cognition and Loneliness in Older Adults**

**(author names withheld for blind review)**

**Supplementary Sensitivity Analyses Based on Randomly Selected Subsamples**

Following the a priori power analysis, the study recruited a sample larger than the minimum required sample size. As clarified in the main text, although oversampling was used to improve statistical precision, no statistical analyses were conducted before the completion of data collection. To further examine whether the main findings depended on the larger final sample, we conducted supplementary sensitivity analyses based on randomly selected subsamples. Specifically, 150 participants were randomly selected from each age group (young-old and old-old), and the key statistical analyses were repeated using these reduced samples. The results revealed patterns that were highly similar to those observed in the full sample, indicating that the main findings were not driven solely by the larger overall sample size. Detailed results are presented below.

**Descriptives and Correlations**

Table S1 lists descriptive statistics and correlations among main variables separately in young-old and old-old. For young-old, SM abuse was correlated with gender (marginally significant; *r* = -.142, *p* = .046, one-tailed) and worse social cognition (SM abuse: *r* = -.219, *p* = .009). For old-old, however, SM use was related to worse social cognition (*r* = -.108, *p* = .093), lower loneliness (*r* = -.186, *p* = .027), and more social support (*r* = .194, *p* = .022), whereas SM abuse was correlated with worse cognitive functioning (*r* = -.263, *p* = .002), lower social cognition (*r* = -.178, *p* = .037), and higher loneliness (*r* = .274, *p* = .001). Overall, SM abuse, but not SM use, had a negative relation with older adults’ cognitive functioning, social cognition and loneliness, with those over 65 years old particularly vulnerable.

**Table S1**

*Descriptive Statistics and Correlations Among Main Variables in Young-Old (Above the Diagonal) and Old-Old (Below the Diagonal)*

|  | Young-old  (*n* = 150) | |  | Old-old  (*n* = 150) | |  | Correlations | | | | | | | | | |
| --- | --- | --- | --- | --- | --- | --- | --- | --- | --- | --- | --- | --- | --- | --- | --- | --- |
|  | *M* | *SD* |  | *M* | *SD* |  | 1 | 2 | 3 | 4 | 5 | 6 | 7 | 8 | 9 |  |
| 1. Age | 61.90 | 1.52 |  | 69.94 | 4.32 |  | – | -.087 | .091 | -.105 | .001 | -.006 | .034 | .012 | .002 |  |
| 2. Gender | – | – |  | – | – |  | .005 | – | .004 | .117 | **-.161^†^** | .015 | -.073 | -.120 | **-.142^†^** |  |
| 3. Education | 3.20 | 1.43 |  | 3.19 | 1.47 |  | .092 | -.052 | – | .042 | -.040 | -.035 | .054 | .084 | .099 |  |
| 4. Cognitive Functioning | .026 | .679 |  | -.022 | .604 |  | -.065 | -.035 | .157 | – | **.220^b^** | -.114 | .072 | .059 | -.071 |  |
| 5. Social Cognition | 25.64 | 4.40 |  | 25.82 | 5.01 |  | -.016 | .083 | .010 | **.277^c^** | – | .017 | -.096 | -.067 | **-.219^b^** |  |
| 6. Loneliness | 2.55 | .793 |  | 2.33 | .771 |  | -.035 | .004 | -.010 | **-.243^b^** | -.042 | – | **-.588^c^** | -.129 | .083 |  |
| 7. Social Support | 13.24 | 5.64 |  | 14.44 | 5.73 |  | .075 | **-.194^a^** | .073 | .060 | .045 | **-.484^c^** | – | .072 | -.039 |  |
| 8. SM Use | 2.71 | 1.18 |  | 2.82 | 1.19 |  | **-.226^b^** | .035 | -.017 | .056 | **-.186^a^** | -.090 | **.194^a^** | – | **.378^c^** |  |
| 9. SM Abuse | 1.87 | .822 |  | 1.80 | .763 |  | -.140 | -.086 | .060 | **-.263^b^** | **-.178^a^** | **.274^c^** | -.027 | **.306^c^** | – |  |

*Note*. Means, standard deviations, and Pearson correlations are presented in the table. Significant correlations are highlighted in bold font. Gender: women = 0, men = 1. In the young-old group, there were 76 women (50.67%), 72 men (48.0%), and 2 responses were missing (1.33%). In the old-old group, there were 88 women (58.67%) and 60 men (40.0%); 2 responses were missing (1.33%). Significant correlations are shown in bold. ^†^*p* < .10, ^a^*p* < .05, ^b^*p* < .01, ^c^*p* < .001.

**SM and Cognitive Functioning**

Next, we examined whether SM use and abuse were significantly associated with cognitive functioning in young-old and old-old adults. Given the correlation between SM abuse and cognitive functioning in the old-old group, we ran two separate hierarchical regressions with cognitive functioning as the dependent variable. The first regression was conducted for the young-old group, and the second for the old-old group. In both analyses, we entered age, gender and education as covariates in Step 1, and entered SM use and abuse simultaneously in Step 2.

Table S2 presents the results of regressions predicting cognitive functioning in the two age groups. For the young-old, cognitive functioning was not predicted by either SM use or SM abuse. For the old-old, however, cognitive functioning was significantly predicted by SM use and abuse together, *∆R^2^ =* .056, *F_change_*(2, 126) = 7.13, *p* = .001. When examining the unique effects of SM use and abuse in the old-old group, SM abuse was a unique predictor of executive functioning, *β =* -.329, *t* = -3.71, *p* < .001, whereas SM use was marginally significant, *β =* .148, *t* = 1.68, *p* = .096. Importantly, this pattern was fully consistent with that reported for the full sample in the main text.

**Table S2**

*Regression Predicting Cognitive Functioning in Young-Old and Old-Old Adults*

|  | Young-old | | |  | Old-old | | |
| --- | --- | --- | --- | --- | --- | --- | --- |
|  | *β* | *p* | *∆R^2^* |  | *β* | *p* | *∆R^2^* |
| *Step 1* |  |  | .029 |  |  |  | .023 |
| Age | -.121 | .155 |  |  | -.051 | .564 |  |
| Gender | .099 | .241 |  |  | .015 | .863 |  |
| Education | .054 | .525 |  |  | .146 | .099 |  |
| *Step 2* |  |  | .009 |  |  |  | .099^b^ |
| Age | -.119 | .162 |  |  | -.072 | .402 |  |
| Gender | .097 | .258 |  |  | -.036 | .671 |  |
| Education | .056 | .510 |  |  | .158 | .062 |  |
| SM Use | .078 | .393 |  |  | .148 | .096 |  |
| SM Abuse | -.092 | .314 |  |  | -.329 | <.001 |  |

*Note*. Age, gender, and education were entered in Step 1 as control variables. *β* represents the standardized regression coefficient, and *∆R²* refers to the additional variance explained by the variables added at each step. ^b^*p* < .01.

**SM and Social Cognition**

We then used two separate regressions (Table S3) to examine whether SM use and abuse were significantly associated with social cognition in the two age groups. In each regression, we entered control variables (age, gender, and education) in Step 1, and then two predictors (SM use and abuse) in Step 2. Building on prior literature indicating the relationship between social cognition and cognitive functioning in older adults and our correlation findings, we also entered cognitive functioning in Step 3 to examine whether the relation between SM use/abuse and social cognition remained after accounting for demographic variables and cognitive functioning. In the young-old group, SM abuse showed a significant negative association with the outcome, *β =* -.249 *t* = -2.79, *p* = .006, which remained significant after accounting for cognitive functioning, *β =* -.227, *t* = -2.60, *p* = .01, whereas SM use was not significantly associated. In contrast, in the old-old group, SM use remained significantly associated with the outcome after controlling for cognitive functioning, *β =* -.247, *t* = -2.81, *p* = .006, while the association with SM abuse was not significant. Cognitive functioning was a strong positive correlate in both groups. Again, these results were identical in pattern to those reported for the full sample in the main text.

**Table S3**

*Regression Predicting Social Cognition in Young-Old and Old-Old Adults*

|  | Young-old | | |  | Old-old | | |
| --- | --- | --- | --- | --- | --- | --- | --- |
|  | *β* | *p* | *∆R^2^* |  | *β* | *p* | *∆R^2^* |
| *Step 1* |  |  | .024 |  |  |  | .017 |
| Age | -.007 | .931 |  |  | .052 | .559 |  |
| Gender | -.152 | .074 |  |  | .122 | .167 |  |
| Education | -.025 | .772 |  |  | .017 | .845 |  |
| *Step 2* |  |  | .059^a^ |  |  |  | .074^b^ |
| Age | -.012 | .888 |  |  | -.006 | .948 |  |
| Gender | -.187 | .028 |  |  | .117 | .179 |  |
| Education | .000 | .999 |  |  | .024 | .782 |  |
| SM Use | .009 | .923 |  |  | -.207 | .023 |  |
| SM Abuse | -.249 | .006 |  |  | -.135 | .135 |  |
| *Step 3* |  |  | .052^b^ |  |  |  | .065^b^ |
| Age | .016 | .845 |  |  | .014 | .870 |  |
| Gender | -.209 | .012 |  |  | .126 | .133 |  |
| Education | -.013 | .871 |  |  | -.019 | .820 |  |
| SM Use | -.010 | .913 |  |  | -.247 | .006 |  |
| SM Abuse | -.227 | .010 |  |  | -.046 | .616 |  |
| Cognitive Functioning | .232 | .005 |  |  | .272 | .002 |  |

*Note*. Age, gender, and education were entered in Step 1 as control variables. *β* represents the standardized regression coefficient, and *∆R²* refers to the additional variance explained by the variables added at each step. ^c^*p* < .001.

**SM Use and Loneliness**

Loneliness and social support were significant correlates of SM use in the old-old group and in the full sample. Therefore, as reported in the main text, our next aim was to examine whether SM use was associated with loneliness through increased social support among old-old adults and across all participants. We used a mediation analysis with the bootstrapping procedure on PROCESS macro (Hayes, 2018; Preacher & Hayes, 2008). Given that age, gender, and education level were correlates of SM use in our results (see Table 1 and Table 2) and previous studies (Janssen et al., 2025), we included these variables as covariates. We generated 5,000 bootstrap samples and computed bias-corrected and accelerated 95% confidence intervals (CI) to assess the indirect effects.

In the old-old group (see Figure S1a), the indirect effect of SM use on loneliness through social support was significant (effect = -.080, *BootSE* = .032, 95% CI [-.147, -.021]), whereas the direct effect of SM use on loneliness was not significant (*β* = -.010, *SE* = .051, *t* = -.203, *p* = .840, 95% CI [-.091, .112]). These results suggest that greater SM use was associated with higher social support, which in turn was associated with lower loneliness, indicating a significant mediating role of social support in the association between SM use and loneliness in old-old adults.

In the full sample, the indirect effect of SM use on loneliness was significant (effect = -.052, *BootSE* = .023, 95% CI = [-.097, -.007]), while the direct effect of SM use on loneliness was not significant (*β* = -.033, *SE* = .034, *t* = -.972, *p* = .332, 95% CI = [-.099, .034], see Figure S1b). The results were consistent with those of the full sample reported in the main text, suggesting that social support played a significant mediating role in the association between SM use and loneliness across all participants.

**Figure 1**

*Mediating Role of Social Support in the Association between Social Media Use and Loneliness Among Old-Old Adults and in the Full Sample*

*Note*. Mediation analyses were carried out after controlling for age, gender, and education. Panel (a) presents the mediation model for the old-old group, and panel (b) presents the mediation model for the full sample. Mediation analysis was not conducted for the young-old group because loneliness, social media use, and social support were not significantly correlated in that group. ns = not significant, ^*^*p* < .05, ^**^*p* < .01, ^***^*p* < .001.

Given the significant correlations among SM use, loneliness, and SM abuse among old-old group and in the full sample, we also examined whether SM use and loneliness predicted unique variance in SM abuse among old-old adults and in all participants (see Table S4). Our goal was to explore the factors that might contribute to older adults’ SM abuse. Age, gender, and education were entered as control variables, and SM use and loneliness were centered and entered into a regression along with the SM use × loneliness interaction.

Among the old-old adults, SM use and loneliness showed independent associations with SM abuse, whereas the SM use × loneliness interaction was marginally significant. A similar pattern was observed in the full sample, in which SM use and loneliness were unique predictors of SM abuse and the interaction term was also marginally significant. Together, these findings suggest that loneliness may moderate the association between SM use and SM abuse, such that the positive association between SM use and SM abuse was stronger at higher levels of loneliness. This was again consistent with the pattern reported for the full sample in the main text.

**Table S4**

*Regression Predicting Older Adults’ Social Media Abuse Among Old-Old Adults and in All Participants*

|  | *B* | *SE* | *β* | *t* | *p* |
| --- | --- | --- | --- | --- | --- |
| *Old-old adults* |  |  |  |  |  |
| Age | -.013 | .009 | -.084 | -1.50 | .135 |
| Gender | -.189 | .086 | -.122 | -2.18 | .030 |
| Education | .032 | .029 | .061 | 1.10 | .273 |
| SM Use | .279 | .047 | .333 | 5.94 | <.001 |
| Loneliness | .148 | .042 | .197 | 3.52 | <.001 |
| SM Use × Loneliness | .085 | .047 | .100 | 1.79 | .074 |
| All Variables: Total *R*^2^ |  |  |  |  | .182 |
| *All participants* |  |  |  |  |  |
| Age | -.017 | .014 | -.100 | -1.21 | .229 |
| Gender | -.215 | .117 | -.147 | -1.83 | .069 |
| Education | .026 | .039 | .055 | .673 | .502 |
| SM Use | .240 | .064 | .305 | 3.73 | <.001 |
| Loneliness | .194 | .058 | .271 | 3.37 | .001 |
| SM Use × Loneliness | .115 | .067 | .141 | 1.72 | .088 |
| All Variables: Total *R*^2^ |  |  |  |  | .203 |

*Note*. *B, SE, β, t* and *p* values are reported with all predictors simultaneously entered into the regression equation. *ΔR^2^* refers to the proportion of independent variance accounted for by each variable in the model.
